# The Sufferings of Christ

**Published:** 1887-09-24

**Authors:** 


### Words of Consolation.

[Communications for this column should be addressed, "Chaplain," care of the Editor of The Hospital, who will gratefully welcome any suggestions or inquiries from matrons, nurses, and the staff generally.]

### LI.—The Sufferings of Christ.

(*For Reading to the Sick.*)

There is yet another point to be considered before we leave the Garden of Gethsemane. Our Lord evidently suffered from some grievous assault upon the Human will, which, since He is truly Man, must have been as liable to temptation to swerve from the Divine decrees as our own. There are indications of His giving up His will to the Father's and becoming completely resigned, yet not without great agony. The struggle seems to have been waged with some terribly strong temptation to decline the cup which the Father wished Him to drink. The temptation was similar to the one first presented to Him in the wilderness, when Satan would have induced Him to renounce the fast ordained by the Higher will. Of course our Blessed Lord's mind was made up long before He became incarnate. The first resolve, "Lo, I come to do Thy will, O God," had been registered in heaven. But when He took upon Him human nature, with its weakness, He often needed to strengthen that resolve during His trials. The object of Satan was to hinder His obedience: and therefore temptation was concentrated, probably much oftener than is revealed, upon the human will, which naturally shrank from suffering. "Not my will, but Thine, be done. And there appeared an angel unto Him from heaven strengthening Him" (Luke xxii, 42, 43). We know that the higher—that is the Divine—will in Christ triumphed. We know how the triumph was the signal for His rising for the last time, and going forth perfectly calm and tranquil, to meet the traitor's band, with His face set as a flint to endure unto the end.

Those who have given themselves, as they think and honestly believe, to the service of God are astonished sometimes at the discovery of how much that yet remains of their wills to offer afresh. There may be submission without resignation. Resignation is the higher grace of the two. Take the nearest illustration possible. We might submit to be led away by a band of men, and be subject to bonds, and to the most shameful treatment conceivable; and because we could see no possible way out of this we would make no resistance, nor any attempt at escape. This need not be resignation. It would not be choosing such circumstances, because God willed us to suffer. We should not be content, because we should escape if we could, even if we knew that all this was ordained. So it is with many trials ordained, such as sickness, home crosses, conditions of life which seem unsuitable; we may submit, but we would get out of the circumstances and surroundings if we could. This is not resignation. Resignation is the entire and absolute triumph of the will of God in us. First must come the acceptance of the circumstances ordained, then the choosing them, and preferring them by an effort of the will, because such circumstances are recognised as best, and therefore happiest, as being the expression of the Divine will. "Submissive would I still reply." But there is in many cases a long pause, much agony, many a struggle before the human will is completely resigned, and the answer really given—"Thy will be done."

(*To be continued.*)

